# Adapting patient and public involvement in patient‐oriented methods research: Reflections in a Canadian setting during COVID‐19

**DOI:** 10.1111/hex.13387

**Published:** 2021-11-12

**Authors:** Jenny Leese, Leana Garraway, Linda Li, Nelly Oelke, Martha MacLeod

**Affiliations:** ^1^ School of Epidemiology and Public Health University of Ottawa Ottawa Canada; ^2^ Centre for Implementation Research The Ottawa Hospital Research Institute Ottawa Canada; ^3^ Arthritis Research Canada Vancouver British Columbia Canada; ^4^ Health Research Institute University of Northern British Columbia Prince George British Columbia Canada; ^5^ Department of Physical Therapy University of British Columbia Vancouver British Columbia Canada; ^6^ School of Nursing University of British Columbia Okanagan British Columbia Canada; ^7^ School of Nursing University of Northern British Columbia Prince George British Columbia Canada

**Keywords:** community participation, COVID‐19, knowledge translation, patient and public involvement, patient‐oriented

## Abstract

**Background:**

Processes of the patient and public involvement (PPI) in health research shifted quickly during 2020. Faced with large‐scale issues, such as the COVID‐19 pandemic, the need to adapt processes of PPI to uphold commitments to nurturing the practice of ‘nothing about us without us’ in research has been urgent and profound. We describe how processes of PPI in research on patient‐oriented methods of knowledge translation and implementation science were adapted by four teams in a Canadian setting.

**Methods:**

As part of an ongoing quality improvement self‐study to enhance PPI within these teams, team members shared their experiences of PPI in the context of this pivotal year during interviews and facilitated discussions. Drawing on these experiences, we outline challenges and reflections for adapting processes of PPI in health research on methods in times of urgency, conflict and fast‐moving change.

**Discussion:**

Our reflections offer insight into common issues encountered across teams that may be amplified during times of rapid change, including handling change and uncertainty, sustaining relationship‐building and hearing differing perspectives in processes of PPI.

**Conclusion:**

These learnings present an opportunity to help others active in or planning patient‐oriented methods research to reflect on the changing nature of PPI and how to adapt PPI processes in response to turbulent situations in the future.

## Patient and Public Contributions

The key reflections presented draw heavily from perspectives shared by eight patient and public partners in interviews and facilitated discussions (the conduct and analysis of data in the quality improvement self‐study).

## 1 INTRODUCTION

In response to recent historic events, such as the coronavirus disease 2019 (COVID‐19) pandemic, approaches to patient and public involvement (PPI) are shifting. PPI involves doing research with, or by, the public rather than to, about, or for the public.^1^ The term ‘public’ refers broadly to patients, potential patients, carers and people who use health and social care services as well as people from organizations that represent people who use these services.^1^ At the heart of PPI is a core value of social justice, shaped by wider societal developments towards realizing citizen empowerment. Requirements for PPI in clinical research by funders in the United Kingdom, the United States and Canada indicate a commitment and aspiration to involve patients and the public, thereby delivering high‐quality research that is meaningful to those it stands to impact most.^2^, ^3^, ^4^ Support for PPI, specifically in patient‐oriented methodological research on KT and implementation science (IS), is growing but in its infancy.^5^ For most, PPI in KT‐IS methods research is not commonplace.

The full impact of COVID‐19 on practices of PPI is not yet known. Academic institutions and community organizations face challenges (e.g., quick transitions, changing priorities, financial shortfalls, increased needs from existing clients) in operating within the rapidly altered landscape, which has the potential to jeopardize advances in PPI.^6^, ^7^ Unfortunately, some early reports indicate disruption and reduction in PPI.^8^, ^9^, ^10^ This is at a time when advancements in PPI are still critical, if not even more so because disparities in health outcomes among communities least likely to be involved in PPI have been exacerbated by COVID‐19.^11^, ^12^, ^13^ Patient and public partners continue to have a right to be involved in the conduct, management and governance of any publicly funded research if they choose.

In this viewpoint, we contribute to a small but growing literature that aims to support research teams to uphold commitments to nurturing PPI across all communities through times of fast‐moving change.^9^, ^10^, ^14^, ^15^, ^16^ Although training and tools developed pre‐2020 exist to support practices of PPI, little evidence is currently available on how to adapt and sustain these practices in ways that sensitively attend to the current context.^1^, ^17^ At this opportune time in the midst of the ongoing COVID‐19 pandemic and its impact on long‐standing health inequities, we aim to stimulate readers' further discussion and inquiry into PPI. We share key reflections on how to adapt and sustain PPI in patient‐oriented methods research informed by perspectives shared in a Canada‐based self‐study.

## 2 A SELF‐STUDY OF PPI IN PATIENT‐ORIENTED METHODS RESEARCH

Across Canada, a Canadian Institutes of Health Research's national initiative, Strategy for Patient‐Oriented Research (SPOR), funds 11 provincial SUPPORT (SUpport for People and Patient‐Oriented Research) Units that aim to support partnerships in patient‐oriented research between academics and others, including patients and the public.^18^ In British Columbia, the SUPPORT Unit has uniquely created six Methods Clusters, which have the mandate to advance methodological research in specific areas by supporting investigator‐initiated projects. The Knowledge Translation‐Implementation Science (KT‐IS) Methods Cluster has funded five projects to date (https://bcsupportunit.ca/about/methods-clusters/knowledge-translation-and-implementation-science). Each project team is diverse in its composition and process of PPI. Each team also has a separate focus, including studying consensus methods in integrated KT to promote patient‐oriented research, a hermeneutic approach to IS, the creation of an online set of systems‐thinking tools for community groups, the development of an online portal for citizen science, and using documentary as a method of KT to reach the ‘sandwich generation’. All are led by senior researchers with long histories of PPI and involve at least one patient partner.

**Figure 1:**
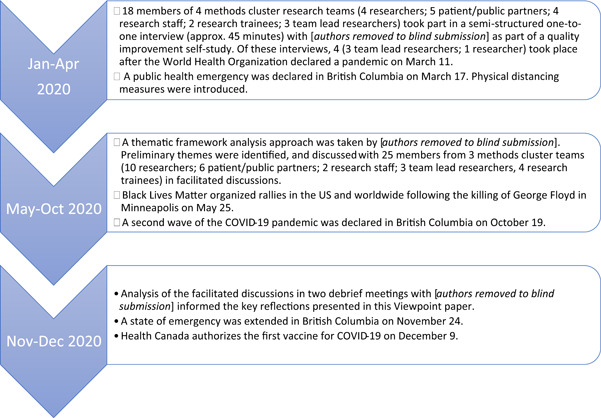
Timeline of our self‐study on patient and public involvement in methods research alongside historic events of 2020

In 2019, we began a quality improvement self‐study with the project teams to gain a better understanding of what it means for patients and the public to partner in research on methods. We conceptualized self‐study as a methodology for studying professional practice that was self‐initiated, collaborative and aimed towards improvement through learning from experience.^19^ The original purpose of the self‐study was to provide the project teams with the opportunity to engage in collaborative reflection on ways of working through common issues encountered in PPI in their methodological research. Informed by the Alberta Innovates Ethics Screening Tool (https://albertainnovates.ca/programs/arecci/), it was determined the self‐study was a low‐risk quality improvement study that did not need research ethics board review. At the design stage of the self‐study, we did not anticipate COVID‐19 and its impact, nor that the self‐study would come to offer insight into how to adapt PPI during times of turbulent social change. Figure 1 provides a timeline of how the self‐study unfolded alongside major events of 2020.

As part of the self‐study, 18 members of four project teams (4 researchers; 5 patient/public partners; 4 research staff; 2 research trainees; 3 project leads) took part in one‐to‐one interviews between January and April 2020. One project team chose not to participate in the interviews due to team priorities before and during the COVID‐19 pandemic; however, the team's project lead provided regular updates on their activities and contributed to overall self‐study reflections. Each interviewer (J. L., L. G.) had over 10 years of experience in conducting qualitative interviews and neither were members of project teams at the BC SUPPORT Unit. Interview participants were asked to describe their experiences of engagement, including any successes, surprises or tensions. Twenty‐five project team members (10 researchers; 6 patient/public partners; 2 research staff; 4 trainees; 3 team lead researchers) then took part in a reflective discussion informed by the interviews in September–October 2020. Two meetings took place in November–December 2020 among authors with a purpose to discuss further theme development in light of perspectives shared during facilitated discussions. During these meetings, authors reflected on how project teams adapted their PPI over the turbulent year. These reflections were informed by ongoing analysis of interview data and perspectives shared during the facilitated discussions, as well as first‐hand experiences of L. L., M. M. and N. O. as project team members. Themes from the ongoing self‐study will be published separately. Reflections from the debrief meetings are presented here to offer early empirical insight for others to consider when aiming to optimize PPI through times of turbulence.

## 3 KEY REFLECTIONS AND DISCUSSION

### 3.1 On handling change and uncertainty

Given the little evidence available to guide PPI in studies of KT‐IS methods, many team members identified that they had been feeling out of their comfort zone at various stages of their involvement before the COVID‐19 pandemic was announced. Although many team members had extensive experience of PPI in research, few had been involved in PPI in research specifically on KT‐IS methods before. At the beginning of the COVID‐19 pandemic, projects in the Methods Cluster halted immediately. Faced with this unexpected interruption, team members relied on approaches they used prepandemic to attend to change and uncertainty COVID‐19 brought about.

One approach identified to attend to change and uncertainty was remaining flexible. Before COVID‐19, plans had often been adapted in response to ‘uncontrollable’ aspects involved in exploring how to do partnered research in KT‐IS methods. Adapting to change and uncertainty with kindness, empathy and patience for other team members as well as themselves was articulated as critical among project team members, to ease feelings of frustration, confusion or guilt that could arise. The role of the project lead was also identified as crucial to providing ‘good orientation’ during times of uncertainty and change. This involved timely check‐in conversations with team members to ask open‐ended questions about expectations and discuss changes to project aims and/or designs due to external contextual demands on team members.

Similar approaches are also supported in the emerging literature. For example, the value of placing a renewed focus on empathy, humanism, honesty and humility is highlighted by Carson et al.^9^ Also, Opara et al. agree that, given that the nature of the COVID‐19 pandemic is dynamic and fast‐changing, frequent and systematic check‐ins with the patient and public partners to re‐evaluate needs and research directions may be warranted.^14^

### 3.2 On sustaining relationship‐building

Many project team members had already built relationships with each other before joining their respective projects, with some having research partnerships that had developed over years. The benefits of forming the project teams with established relationships were recognized among team members before COVID‐19, particularly because easier social connections and trust that had been built over time helped to mitigate feelings of unease during times of uncertainty and change. Having established these trusting relationships was a particular advantage to sustaining relationship‐building after the outbreak of COVID‐19 because they allowed team members to be more open and responsive to each other's needs in the fast‐changing context.

Before COVID‐19, sustaining relationships had involved taking opportunities to engage in conversation or ‘chit‐chat’ that was non‐project‐related with other team members. The project lead created these opportunities during regular in‐person team meetings or by making themselves available outside of these meetings. A research staff member also described meeting a patient/public partner over coffee to connect on a more informal level while discussing their project. During the COVID‐19 pandemic, the need to evolve these usual methods for sustaining relationships was immediate, especially with physical distancing measures and no in‐person activities. Leveraging online communication platforms was welcomed, particularly by a patient partner who had used technology regularly before COVID‐19 to join team meetings. He emphasized too, however, there were sometimes technical glitches, or had difficulty sharing documents or ideas when brainstorming with other attendees.

Others also reworked quickly with large teams to continue PPI during the COVID‐19 pandemic, supported by long‐standing relationships.^20^, ^21^, ^22^ Much support has also been given to exploring new approaches that enable partners to sustain relationships through online communication.^10^, ^16^ Others also experienced some challenges (e.g., feelings of apprehension) in adapting to sustain partnerships using online platforms during the COVID‐19 pandemic.^9^, ^10^, ^14^ Based on the experiences of patient and public partners they had been working with during the COVID‐19 pandemic, Chew‐Graham recommends making contact with partners before virtual meetings to offer technical support and help them to feel comfortable using online platforms available.^10^ Opara et al. suggest cohosting virtual meetings may be helpful, particularly for community partners who may not readily have access to virtual meeting platforms, as an example of creatively leveraging resources.^14^

More can also be learned about how online communication could provide opportunities to build new relationships with additional partners who previously would not have been able to travel to meetings in person. Hausmann et al., for example, describe how, in a few short months, social media platforms enabled researchers and patient and public partners (including over 100 patient support organizations) to partner during the COVID‐19 pandemic as part of the COVID‐19 Global Rheumatology Alliance.^23^ We also acknowledge that some patients and public partners may not have access to the internet or technology to be able to connect remotely. Further research is therefore needed to learn more about how to successfully sustain relationships in PPI with and without technology during times of fast‐moving change.

### 3.3 On hearing differing perspectives

Before the outbreak of COVID‐19, project team members identified hearing differing perspectives as a vital component of successful PPI. The importance of hearing others' perspectives in the process of establishing shared goals and reaching mutual benefit was commonly highlighted. A key benefit of hearing differing perspectives shared by patient and public partners was that it kept discussions focused or ‘grounded’ in the real‐world significance of the studies, which was particularly important given the seemingly more abstract nature of research on methods.

During facilitated discussions of the self‐study, project team members revisited conversations on what it meant to hear differing perspectives in PPI in 2020. These discussions were particularly prompted by ongoing issues of social justice that gained increased attention through the Black Lives Matter (BLM) movement and efforts of Truth and Reconciliation with Indigenous Peoples. The COVID‐19 pandemic had intensified the spotlight on existing inequalities and the value of engaging with patient and public partners most at‐risk, including Indigenous, Black, Latino and other Minority Ethnic partners.^11^, ^12^, ^13^ The BLM movement also intensified widespread recognition of persistent disparities in health outcomes by race, ethnicity, gender identity and sexual orientation. Moreover, a report entitled, *In Plain Sight*, was released in November 2020 describing widespread systemic racism and discrimination against Indigenous peoples in the British Columbia health care system, perpetuating the existing inequities.^24^ Patient and public partners reassessed their place as project team members and requested team lead researchers develop new ways of hearing differing perspectives from partners most at‐risk from these inequities.

Others have highlighted that PPI has been criticized for not sufficiently including patient and public partners from Indigenous, Black, Latino and other Minority Ethnic communities.^9^, ^15^, ^16^ A rapid‐cycle priority identification process conducted by the Canadian Institutes of Health Research to inform Canada's research response to COVID‐19 also identified supporting the health of Indigenous Peoples and other at‐risk populations (e.g., people who are homeless, incarcerated or living in poverty) as a key priority for PPI.^25^ Further research is needed to explore paths forward to hearing differing perspectives in PPI with communities most at‐risk, particularly in the midst of turbulence. The importance of using inclusive language in exploring these paths forward has also been emphasized.^15^, ^26^ We highlight too, the importance of avoiding tokenistic efforts by fostering authentic partnerships over time while acknowledging the difficulties of building trust given legacies of oppression.

## 4 CONCLUSIONS

Our reflections contribute to the growing literature that can support meaningful PPI practices to adapt and persist in turbulent times of fast‐moving change. Recent historic events disrupted many aspects of PPI. It is imperative researchers take advantage of the opportunities this disruption has brought to improve PPI practices so that all patient and public partners are meaningfully involved in shaping the future of their health through involvement in research if they choose.

## CONFLICT OF INTERESTS

The authors declare that there are no conflict of interests.

## AUTHOR CONTRIBUTIONS

All authors contributed to the conceptualization of this manuscript. Jenny Leese led the initial draft with substantial contributions from Martha MacLeod. All authors commented and contributed to the revision of the final version. Jenny Leese and Leana Garraway are research associates with experience in qualitative research and patient and public involvement in research.

## Data Availability

Research data not applicable to this Viewpoint article.
